# Supplementary material for: Pyruvate Dehydrogenase Kinase 1 inhibition mediated oxidative phosphorylation enhancement in cartilage promotes osteoarthritis progression
Source: BMC Musculoskelet Disord. 2023 Jul 20;24:597. doi: 10.1186/s12891-023-06585-6 (PMC10357736; doi:10.1186/s12891-023-06585-6)
Supplement: Supplementary file 1 — Additional file 1. [file 12891_2023_6585_MOESM1_ESM.zip › MF.Gsea.1653627316242/GOMF_DNA_BINDING_TRANSCRIPTION_ACTIVATOR_ACTIVITY.html]

Details for gene set GOMF\_DNA\_BINDING\_TRANSCRIPTION\_ACTIVATOR\_ACTIVITY[GSEA]

|  || Dataset | 55457\_collapsed\_to\_symbols.cls.cls #OA\_versus\_normal.cls.cls #OA\_versus\_normal\_repos |
| Phenotype | cls.cls#OA\_versus\_normal\_repos |
| Upregulated in class | normal |
| GeneSet | GOMF\_DNA\_BINDING\_TRANSCRIPTION\_ACTIVATOR\_ACTIVITY |
| Enrichment Score (ES) | -0.30183733 |
| Normalized Enrichment Score (NES) | -1.5491912 |
| Nominal p-value | 0.0 |
| FDR q-value | 0.1582465 |
| FWER p-Value | 1.0 |
Table: GSEA Results Summary

  

Fig 1: Enrichment plot: GOMF\_DNA\_BINDING\_TRANSCRIPTION\_ACTIVATOR\_ACTIVITY      
 Profile of the Running ES Score & Positions of GeneSet Members on the Rank Ordered List

  

| SYMBOL | TITLE | RANK IN GENE LIST | RANK METRIC SCORE | RUNNING ES | CORE ENRICHMENT || 1 | ZIC1 | Zic family member 1 [Source:HGNC Symbol;Acc:HGNC:12872] | 0 | 1.459 | 0.0124 | No |
| 2 | NDN | "necdin, MAGE family member [Source:HGNC Symbol;Acc:HGNC:7675]" | 191 | 0.827 | 0.0042 | No |
| 3 | HHEX | hematopoietically expressed homeobox [Source:HGNC Symbol;Acc:HGNC:4901] | 270 | 0.774 | 0.0045 | No |
| 4 | SMAD2 | SMAD family member 2 [Source:HGNC Symbol;Acc:HGNC:6768] | 275 | 0.768 | 0.0107 | No |
| 5 | PAX6 | paired box 6 [Source:HGNC Symbol;Acc:HGNC:8620] | 304 | 0.746 | 0.0148 | No |
| 6 | ELF3 | E74 like ETS transcription factor 3 [Source:HGNC Symbol;Acc:HGNC:3318] | 386 | 0.711 | 0.0144 | No |
| 7 | SOX30 | SRY-box transcription factor 30 [Source:HGNC Symbol;Acc:HGNC:30635] | 503 | 0.658 | 0.0107 | No |
| 8 | HOXB7 | homeobox B7 [Source:HGNC Symbol;Acc:HGNC:5118] | 547 | 0.643 | 0.0127 | No |
| 9 | IRF2 | interferon regulatory factor 2 [Source:HGNC Symbol;Acc:HGNC:6117] | 612 | 0.619 | 0.0128 | No |
| 10 | MEIS1 | Meis homeobox 1 [Source:HGNC Symbol;Acc:HGNC:7000] | 625 | 0.613 | 0.0171 | No |
| 11 | NR3C1 | nuclear receptor subfamily 3 group C member 1 [Source:HGNC Symbol;Acc:HGNC:7978] | 674 | 0.599 | 0.0183 | No |
| 12 | HOXC10 | homeobox C10 [Source:HGNC Symbol;Acc:HGNC:5122] | 717 | 0.589 | 0.0200 | No |
| 13 | MYBL2 | MYB proto-oncogene like 2 [Source:HGNC Symbol;Acc:HGNC:7548] | 767 | 0.576 | 0.0209 | No |
| 14 | PBX3 | PBX homeobox 3 [Source:HGNC Symbol;Acc:HGNC:8634] | 858 | 0.553 | 0.0184 | No |
| 15 | ZNF668 | zinc finger protein 668 [Source:HGNC Symbol;Acc:HGNC:25821] | 955 | 0.527 | 0.0152 | No |
| 16 | ESR1 | estrogen receptor 1 [Source:HGNC Symbol;Acc:HGNC:3467] | 984 | 0.522 | 0.0174 | No |
| 17 | NEUROD1 | neuronal differentiation 1 [Source:HGNC Symbol;Acc:HGNC:7762] | 1008 | 0.515 | 0.0200 | No |
| 18 | CREB3 | cAMP responsive element binding protein 3 [Source:HGNC Symbol;Acc:HGNC:2347] | 1036 | 0.510 | 0.0221 | No |
| 19 | TFDP1 | transcription factor Dp-1 [Source:HGNC Symbol;Acc:HGNC:11749] | 1048 | 0.508 | 0.0255 | No |
| 20 | TLX2 | T cell leukemia homeobox 2 [Source:HGNC Symbol;Acc:HGNC:5057] | 1058 | 0.505 | 0.0291 | No |
| 21 | ZNF112 | zinc finger protein 112 [Source:HGNC Symbol;Acc:HGNC:12892] | 1083 | 0.500 | 0.0314 | No |
| 22 | NFYC | nuclear transcription factor Y subunit gamma [Source:HGNC Symbol;Acc:HGNC:7806] | 1104 | 0.495 | 0.0340 | No |
| 23 | SHOX | short stature homeobox [Source:HGNC Symbol;Acc:HGNC:10853] | 1278 | 0.462 | 0.0241 | No |
| 24 | RFX5 | regulatory factor X5 [Source:HGNC Symbol;Acc:HGNC:9986] | 1343 | 0.451 | 0.0228 | No |
| 25 | HOXA1 | homeobox A1 [Source:HGNC Symbol;Acc:HGNC:5099] | 1366 | 0.448 | 0.0249 | No |
| 26 | RFXANK | regulatory factor X associated ankyrin containing protein [Source:HGNC Symbol;Acc:HGNC:9987] | 1383 | 0.445 | 0.0274 | No |
| 27 | HLF | "HLF transcription factor, PAR bZIP family member [Source:HGNC Symbol;Acc:HGNC:4977]" | 1461 | 0.434 | 0.0249 | No |
| 28 | POU4F3 | POU class 4 homeobox 3 [Source:HGNC Symbol;Acc:HGNC:9220] | 1465 | 0.434 | 0.0283 | No |
| 29 | MYBL1 | MYB proto-oncogene like 1 [Source:HGNC Symbol;Acc:HGNC:7547] | 1484 | 0.431 | 0.0306 | No |
| 30 | ETV5 | ETS variant transcription factor 5 [Source:HGNC Symbol;Acc:HGNC:3494] | 1580 | 0.416 | 0.0265 | No |
| 31 | DBP | D-box binding PAR bZIP transcription factor [Source:HGNC Symbol;Acc:HGNC:2697] | 1699 | 0.399 | 0.0205 | No |
| 32 | NR1H3 | nuclear receptor subfamily 1 group H member 3 [Source:HGNC Symbol;Acc:HGNC:7966] | 1728 | 0.394 | 0.0216 | No |
| 33 | CEBPA | CCAAT enhancer binding protein alpha [Source:HGNC Symbol;Acc:HGNC:1833] | 1750 | 0.392 | 0.0232 | No |
| 34 | CARF | calcium responsive transcription factor [Source:HGNC Symbol;Acc:HGNC:14435] | 1792 | 0.385 | 0.0232 | No |
| 35 | ZNF606 | zinc finger protein 606 [Source:HGNC Symbol;Acc:HGNC:25879] | 1801 | 0.384 | 0.0258 | No |
| 36 | RFX4 | regulatory factor X4 [Source:HGNC Symbol;Acc:HGNC:9985] | 1840 | 0.380 | 0.0260 | No |
| 37 | GBX2 | gastrulation brain homeobox 2 [Source:HGNC Symbol;Acc:HGNC:4186] | 1861 | 0.378 | 0.0276 | No |
| 38 | SIX1 | SIX homeobox 1 [Source:HGNC Symbol;Acc:HGNC:10887] | 2011 | 0.360 | 0.0188 | No |
| 39 | NFE2L2 | "nuclear factor, erythroid 2 like 2 [Source:HGNC Symbol;Acc:HGNC:7782]" | 2034 | 0.357 | 0.0200 | No |
| 40 | ZNF345 | zinc finger protein 345 [Source:HGNC Symbol;Acc:HGNC:16367] | 2067 | 0.354 | 0.0205 | No |
| 41 | SALL2 | spalt like transcription factor 2 [Source:HGNC Symbol;Acc:HGNC:10526] | 2072 | 0.354 | 0.0232 | No |
| 42 | SOX9 | SRY-box transcription factor 9 [Source:HGNC Symbol;Acc:HGNC:11204] | 2089 | 0.351 | 0.0249 | No |
| 43 | FLI1 | "Fli-1 proto-oncogene, ETS transcription factor [Source:HGNC Symbol;Acc:HGNC:3749]" | 2092 | 0.350 | 0.0277 | No |
| 44 | GRHL2 | grainyhead like transcription factor 2 [Source:HGNC Symbol;Acc:HGNC:2799] | 2111 | 0.348 | 0.0292 | No |
| 45 | PITX1 | paired like homeodomain 1 [Source:HGNC Symbol;Acc:HGNC:9004] | 2263 | 0.331 | 0.0199 | No |
| 46 | FEZF2 | FEZ family zinc finger 2 [Source:HGNC Symbol;Acc:HGNC:13506] | 2278 | 0.329 | 0.0216 | No |
| 47 | ZNF780B | zinc finger protein 780B [Source:HGNC Symbol;Acc:HGNC:33109] | 2363 | 0.319 | 0.0176 | No |
| 48 | ISL1 | ISL LIM homeobox 1 [Source:HGNC Symbol;Acc:HGNC:6132] | 2373 | 0.318 | 0.0196 | No |
| 49 | HOXA10 | homeobox A10 [Source:HGNC Symbol;Acc:HGNC:5100] | 2408 | 0.313 | 0.0195 | No |
| 50 | ZNF629 | zinc finger protein 629 [Source:HGNC Symbol;Acc:HGNC:29008] | 2447 | 0.308 | 0.0191 | No |
| 51 | ZNF750 | zinc finger protein 750 [Source:HGNC Symbol;Acc:HGNC:25843] | 2449 | 0.308 | 0.0216 | No |
| 52 | HNF4G | hepatocyte nuclear factor 4 gamma [Source:HGNC Symbol;Acc:HGNC:5026] | 2509 | 0.302 | 0.0195 | No |
| 53 | PAX5 | paired box 5 [Source:HGNC Symbol;Acc:HGNC:8619] | 2546 | 0.298 | 0.0191 | No |
| 54 | PHOX2A | paired like homeobox 2A [Source:HGNC Symbol;Acc:HGNC:691] | 2789 | 0.271 | 0.0021 | No |
| 55 | MEIS2 | Meis homeobox 2 [Source:HGNC Symbol;Acc:HGNC:7001] | 2829 | 0.267 | 0.0013 | No |
| 56 | EGR4 | early growth response 4 [Source:HGNC Symbol;Acc:HGNC:3241] | 2830 | 0.267 | 0.0035 | No |
| 57 | ZNF43 | zinc finger protein 43 [Source:HGNC Symbol;Acc:HGNC:13109] | 2860 | 0.264 | 0.0034 | No |
| 58 | ESRRG | estrogen related receptor gamma [Source:HGNC Symbol;Acc:HGNC:3474] | 2874 | 0.263 | 0.0046 | No |
| 59 | ZBED1 | zinc finger BED-type containing 1 [Source:HGNC Symbol;Acc:HGNC:447] | 2912 | 0.259 | 0.0039 | No |
| 60 | GATA4 | GATA binding protein 4 [Source:HGNC Symbol;Acc:HGNC:4173] | 3009 | 0.249 | -0.0017 | No |
| 61 | NFATC1 | nuclear factor of activated T cells 1 [Source:HGNC Symbol;Acc:HGNC:7775] | 3027 | 0.247 | -0.0009 | No |
| 62 | NFATC3 | nuclear factor of activated T cells 3 [Source:HGNC Symbol;Acc:HGNC:7777] | 3079 | 0.242 | -0.0030 | No |
| 63 | PLAG1 | PLAG1 zinc finger [Source:HGNC Symbol;Acc:HGNC:9045] | 3095 | 0.240 | -0.0021 | No |
| 64 | CLOCK | clock circadian regulator [Source:HGNC Symbol;Acc:HGNC:2082] | 3231 | 0.227 | -0.0110 | No |
| 65 | ZNF175 | zinc finger protein 175 [Source:HGNC Symbol;Acc:HGNC:12964] | 3266 | 0.224 | -0.0118 | No |
| 66 | HOXB5 | homeobox B5 [Source:HGNC Symbol;Acc:HGNC:5116] | 3326 | 0.219 | -0.0146 | No |
| 67 | ELK1 | ETS transcription factor ELK1 [Source:HGNC Symbol;Acc:HGNC:3321] | 3357 | 0.216 | -0.0152 | No |
| 68 | ONECUT1 | one cut homeobox 1 [Source:HGNC Symbol;Acc:HGNC:8138] | 3443 | 0.207 | -0.0202 | No |
| 69 | ZNF107 | zinc finger protein 107 [Source:HGNC Symbol;Acc:HGNC:12887] | 3471 | 0.205 | -0.0207 | No |
| 70 | ARNT2 | aryl hydrocarbon receptor nuclear translocator 2 [Source:HGNC Symbol;Acc:HGNC:16876] | 3512 | 0.201 | -0.0221 | No |
| 71 | MYB | "MYB proto-oncogene, transcription factor [Source:HGNC Symbol;Acc:HGNC:7545]" | 3553 | 0.198 | -0.0237 | No |
| 72 | ZNF544 | zinc finger protein 544 [Source:HGNC Symbol;Acc:HGNC:16759] | 3611 | 0.192 | -0.0266 | No |
| 73 | RFXAP | regulatory factor X associated protein [Source:HGNC Symbol;Acc:HGNC:9988] | 3624 | 0.191 | -0.0259 | No |
| 74 | PRRX1 | paired related homeobox 1 [Source:HGNC Symbol;Acc:HGNC:9142] | 3657 | 0.189 | -0.0269 | No |
| 75 | PAX8 | paired box 8 [Source:HGNC Symbol;Acc:HGNC:8622] | 3680 | 0.187 | -0.0270 | No |
| 76 | RORB | RAR related orphan receptor B [Source:HGNC Symbol;Acc:HGNC:10259] | 3705 | 0.185 | -0.0274 | No |
| 77 | OSR2 | odd-skipped related transciption factor 2 [Source:HGNC Symbol;Acc:HGNC:15830] | 3715 | 0.184 | -0.0265 | No |
| 78 | ZIC3 | Zic family member 3 [Source:HGNC Symbol;Acc:HGNC:12874] | 3736 | 0.182 | -0.0266 | No |
| 79 | ZNF33B | zinc finger protein 33B [Source:HGNC Symbol;Acc:HGNC:13097] | 3775 | 0.179 | -0.0281 | No |
| 80 | HOXB2 | homeobox B2 [Source:HGNC Symbol;Acc:HGNC:5113] | 3884 | 0.166 | -0.0353 | No |
| 81 | PBX1 | PBX homeobox 1 [Source:HGNC Symbol;Acc:HGNC:8632] | 3887 | 0.166 | -0.0341 | No |
| 82 | RELA | "RELA proto-oncogene, NF-kB subunit [Source:HGNC Symbol;Acc:HGNC:9955]" | 3942 | 0.161 | -0.0370 | No |
| 83 | NR1I2 | nuclear receptor subfamily 1 group I member 2 [Source:HGNC Symbol;Acc:HGNC:7968] | 3956 | 0.160 | -0.0367 | No |
| 84 | RUNX2 | RUNX family transcription factor 2 [Source:HGNC Symbol;Acc:HGNC:10472] | 3993 | 0.157 | -0.0383 | No |
| 85 | MYF6 | myogenic factor 6 [Source:HGNC Symbol;Acc:HGNC:7566] | 4057 | 0.150 | -0.0420 | No |
| 86 | SMAD3 | SMAD family member 3 [Source:HGNC Symbol;Acc:HGNC:6769] | 4061 | 0.150 | -0.0410 | No |
| 87 | HOXA5 | homeobox A5 [Source:HGNC Symbol;Acc:HGNC:5106] | 4062 | 0.150 | -0.0397 | No |
| 88 | SOX1 | SRY-box transcription factor 1 [Source:HGNC Symbol;Acc:HGNC:11189] | 4065 | 0.150 | -0.0386 | No |
| 89 | MEF2C | myocyte enhancer factor 2C [Source:HGNC Symbol;Acc:HGNC:6996] | 4218 | 0.135 | -0.0496 | No |
| 90 | ZNF493 | zinc finger protein 493 [Source:HGNC Symbol;Acc:HGNC:23708] | 4267 | 0.131 | -0.0523 | No |
| 91 | ZNF639 | zinc finger protein 639 [Source:HGNC Symbol;Acc:HGNC:30950] | 4274 | 0.131 | -0.0517 | No |
| 92 | ZNF226 | zinc finger protein 226 [Source:HGNC Symbol;Acc:HGNC:13019] | 4298 | 0.128 | -0.0524 | No |
| 93 | TFEC | transcription factor EC [Source:HGNC Symbol;Acc:HGNC:11754] | 4306 | 0.128 | -0.0519 | No |
| 94 | TCF21 | transcription factor 21 [Source:HGNC Symbol;Acc:HGNC:11632] | 4321 | 0.127 | -0.0520 | No |
| 95 | IRF6 | interferon regulatory factor 6 [Source:HGNC Symbol;Acc:HGNC:6121] | 4350 | 0.124 | -0.0531 | No |
| 96 | NFYB | nuclear transcription factor Y subunit beta [Source:HGNC Symbol;Acc:HGNC:7805] | 4389 | 0.122 | -0.0551 | No |
| 97 | ATOH1 | atonal bHLH transcription factor 1 [Source:HGNC Symbol;Acc:HGNC:797] | 4428 | 0.119 | -0.0572 | No |
| 98 | NEUROG3 | neurogenin 3 [Source:HGNC Symbol;Acc:HGNC:13806] | 4443 | 0.118 | -0.0573 | No |
| 99 | ZNF225 | zinc finger protein 225 [Source:HGNC Symbol;Acc:HGNC:13018] | 4537 | 0.110 | -0.0638 | No |
| 100 | E2F5 | E2F transcription factor 5 [Source:HGNC Symbol;Acc:HGNC:3119] | 4542 | 0.110 | -0.0632 | No |
| 101 | ZNF395 | zinc finger protein 395 [Source:HGNC Symbol;Acc:HGNC:18737] | 4549 | 0.109 | -0.0627 | No |
| 102 | MYCN | "MYCN proto-oncogene, bHLH transcription factor [Source:HGNC Symbol;Acc:HGNC:7559]" | 4554 | 0.109 | -0.0621 | No |
| 103 | NR1I3 | nuclear receptor subfamily 1 group I member 3 [Source:HGNC Symbol;Acc:HGNC:7969] | 4589 | 0.105 | -0.0639 | No |
| 104 | GCM2 | glial cells missing transcription factor 2 [Source:HGNC Symbol;Acc:HGNC:4198] | 4624 | 0.102 | -0.0658 | No |
| 105 | NFKB1 | nuclear factor kappa B subunit 1 [Source:HGNC Symbol;Acc:HGNC:7794] | 4626 | 0.102 | -0.0650 | No |
| 106 | ONECUT2 | one cut homeobox 2 [Source:HGNC Symbol;Acc:HGNC:8139] | 4647 | 0.100 | -0.0657 | No |
| 107 | DLX5 | distal-less homeobox 5 [Source:HGNC Symbol;Acc:HGNC:2918] | 4700 | 0.096 | -0.0691 | No |
| 108 | SREBF1 | sterol regulatory element binding transcription factor 1 [Source:HGNC Symbol;Acc:HGNC:11289] | 4729 | 0.093 | -0.0705 | No |
| 109 | TBX5 | T-box transcription factor 5 [Source:HGNC Symbol;Acc:HGNC:11604] | 4748 | 0.091 | -0.0712 | No |
| 110 | MITF | melanocyte inducing transcription factor [Source:HGNC Symbol;Acc:HGNC:7105] | 4759 | 0.090 | -0.0712 | No |
| 111 | SOX21 | SRY-box transcription factor 21 [Source:HGNC Symbol;Acc:HGNC:11197] | 4776 | 0.089 | -0.0718 | No |
| 112 | TBX3 | T-box transcription factor 3 [Source:HGNC Symbol;Acc:HGNC:11602] | 4790 | 0.088 | -0.0720 | No |
| 113 | HEYL | hes related family bHLH transcription factor with YRPW motif like [Source:HGNC Symbol;Acc:HGNC:4882] | 4820 | 0.086 | -0.0736 | No |
| 114 | HOXD13 | homeobox D13 [Source:HGNC Symbol;Acc:HGNC:5136] | 4837 | 0.084 | -0.0742 | No |
| 115 | OVOL2 | ovo like zinc finger 2 [Source:HGNC Symbol;Acc:HGNC:15804] | 5017 | 0.071 | -0.0879 | No |
| 116 | IRF4 | interferon regulatory factor 4 [Source:HGNC Symbol;Acc:HGNC:6119] | 5064 | 0.067 | -0.0910 | No |
| 117 | MEOX2 | mesenchyme homeobox 2 [Source:HGNC Symbol;Acc:HGNC:7014] | 5128 | 0.062 | -0.0955 | No |
| 118 | NKRF | NFKB repressing factor [Source:HGNC Symbol;Acc:HGNC:19374] | 5136 | 0.061 | -0.0955 | No |
| 119 | E2F1 | E2F transcription factor 1 [Source:HGNC Symbol;Acc:HGNC:3113] | 5137 | 0.061 | -0.0950 | No |
| 120 | CDX1 | caudal type homeobox 1 [Source:HGNC Symbol;Acc:HGNC:1805] | 5148 | 0.060 | -0.0953 | No |
| 121 | HOXB3 | homeobox B3 [Source:HGNC Symbol;Acc:HGNC:5114] | 5209 | 0.056 | -0.0996 | No |
| 122 | TP73 | tumor protein p73 [Source:HGNC Symbol;Acc:HGNC:12003] | 5222 | 0.055 | -0.1001 | No |
| 123 | FOXC1 | forkhead box C1 [Source:HGNC Symbol;Acc:HGNC:3800] | 5227 | 0.054 | -0.1000 | No |
| 124 | TFCP2 | transcription factor CP2 [Source:HGNC Symbol;Acc:HGNC:11748] | 5273 | 0.050 | -0.1031 | No |
| 125 | TFAP2A | transcription factor AP-2 alpha [Source:HGNC Symbol;Acc:HGNC:11742] | 5379 | 0.041 | -0.1112 | No |
| 126 | MEOX1 | mesenchyme homeobox 1 [Source:HGNC Symbol;Acc:HGNC:7013] | 5384 | 0.041 | -0.1111 | No |
| 127 | TFDP2 | transcription factor Dp-2 [Source:HGNC Symbol;Acc:HGNC:11751] | 5394 | 0.041 | -0.1115 | No |
| 128 | NR1H2 | nuclear receptor subfamily 1 group H member 2 [Source:HGNC Symbol;Acc:HGNC:7965] | 5413 | 0.039 | -0.1126 | No |
| 129 | WT1 | WT1 transcription factor [Source:HGNC Symbol;Acc:HGNC:12796] | 5452 | 0.037 | -0.1153 | No |
| 130 | CDC5L | cell division cycle 5 like [Source:HGNC Symbol;Acc:HGNC:1743] | 5460 | 0.036 | -0.1156 | No |
| 131 | TP53 | tumor protein p53 [Source:HGNC Symbol;Acc:HGNC:11998] | 5511 | 0.032 | -0.1193 | No |
| 132 | LHX2 | LIM homeobox 2 [Source:HGNC Symbol;Acc:HGNC:6594] | 5516 | 0.031 | -0.1194 | No |
| 133 | NR2E3 | nuclear receptor subfamily 2 group E member 3 [Source:HGNC Symbol;Acc:HGNC:7974] | 5530 | 0.030 | -0.1201 | No |
| 134 | STAT5B | signal transducer and activator of transcription 5B [Source:HGNC Symbol;Acc:HGNC:11367] | 5554 | 0.028 | -0.1217 | No |
| 135 | GFI1B | growth factor independent 1B transcriptional repressor [Source:HGNC Symbol;Acc:HGNC:4238] | 5591 | 0.025 | -0.1244 | No |
| 136 | MECOM | MDS1 and EVI1 complex locus [Source:HGNC Symbol;Acc:HGNC:3498] | 5622 | 0.022 | -0.1266 | No |
| 137 | MEF2A | myocyte enhancer factor 2A [Source:HGNC Symbol;Acc:HGNC:6993] | 5628 | 0.022 | -0.1268 | No |
| 138 | HOXA7 | homeobox A7 [Source:HGNC Symbol;Acc:HGNC:5108] | 5634 | 0.021 | -0.1270 | No |
| 139 | SPIB | Spi-B transcription factor [Source:HGNC Symbol;Acc:HGNC:11242] | 5657 | 0.019 | -0.1286 | No |
| 140 | ZNF585B | zinc finger protein 585B [Source:HGNC Symbol;Acc:HGNC:30948] | 5661 | 0.019 | -0.1287 | No |
| 141 | ATMIN | ATM interactor [Source:HGNC Symbol;Acc:HGNC:29034] | 5670 | 0.018 | -0.1292 | No |
| 142 | SOX12 | SRY-box transcription factor 12 [Source:HGNC Symbol;Acc:HGNC:11198] | 5727 | 0.013 | -0.1336 | No |
| 143 | CEBPG | CCAAT enhancer binding protein gamma [Source:HGNC Symbol;Acc:HGNC:1837] | 5734 | 0.013 | -0.1339 | No |
| 144 | SOX11 | SRY-box transcription factor 11 [Source:HGNC Symbol;Acc:HGNC:11191] | 5822 | 0.007 | -0.1408 | No |
| 145 | ZFY | zinc finger protein Y-linked [Source:HGNC Symbol;Acc:HGNC:12870] | 5909 | 0.002 | -0.1477 | No |
| 146 | NHLH1 | nescient helix-loop-helix 1 [Source:HGNC Symbol;Acc:HGNC:7817] | 5998 | -0.004 | -0.1547 | No |
| 147 | BNC1 | basonuclin 1 [Source:HGNC Symbol;Acc:HGNC:1081] | 6023 | -0.006 | -0.1565 | No |
| 148 | EHF | ETS homologous factor [Source:HGNC Symbol;Acc:HGNC:3246] | 6042 | -0.008 | -0.1579 | No |
| 149 | ZNF227 | zinc finger protein 227 [Source:HGNC Symbol;Acc:HGNC:13020] | 6046 | -0.008 | -0.1581 | No |
| 150 | SOX18 | SRY-box transcription factor 18 [Source:HGNC Symbol;Acc:HGNC:11194] | 6053 | -0.008 | -0.1585 | No |
| 151 | FOXL2 | forkhead box L2 [Source:HGNC Symbol;Acc:HGNC:1092] | 6068 | -0.009 | -0.1595 | No |
| 152 | TP63 | tumor protein p63 [Source:HGNC Symbol;Acc:HGNC:15979] | 6274 | -0.025 | -0.1757 | No |
| 153 | SIX3 | SIX homeobox 3 [Source:HGNC Symbol;Acc:HGNC:10889] | 6356 | -0.032 | -0.1818 | No |
| 154 | TCF12 | transcription factor 12 [Source:HGNC Symbol;Acc:HGNC:11623] | 6398 | -0.035 | -0.1848 | No |
| 155 | FOXJ2 | forkhead box J2 [Source:HGNC Symbol;Acc:HGNC:24818] | 6411 | -0.036 | -0.1855 | No |
| 156 | LHX3 | LIM homeobox 3 [Source:HGNC Symbol;Acc:HGNC:6595] | 6495 | -0.042 | -0.1917 | No |
| 157 | FOXO1 | forkhead box O1 [Source:HGNC Symbol;Acc:HGNC:3819] | 6511 | -0.043 | -0.1926 | No |
| 158 | ETV1 | ETS variant transcription factor 1 [Source:HGNC Symbol;Acc:HGNC:3490] | 6581 | -0.048 | -0.1977 | No |
| 159 | GTF2I | general transcription factor IIi [Source:HGNC Symbol;Acc:HGNC:4659] | 6588 | -0.049 | -0.1977 | No |
| 160 | ZBED4 | zinc finger BED-type containing 4 [Source:HGNC Symbol;Acc:HGNC:20721] | 6708 | -0.056 | -0.2068 | No |
| 161 | MAF | MAF bZIP transcription factor [Source:HGNC Symbol;Acc:HGNC:6776] | 6711 | -0.056 | -0.2064 | No |
| 162 | HINFP | histone H4 transcription factor [Source:HGNC Symbol;Acc:HGNC:17850] | 6776 | -0.061 | -0.2110 | No |
| 163 | HOXD3 | homeobox D3 [Source:HGNC Symbol;Acc:HGNC:5137] | 6810 | -0.064 | -0.2131 | No |
| 164 | TFAP4 | transcription factor AP-4 [Source:HGNC Symbol;Acc:HGNC:11745] | 6823 | -0.065 | -0.2135 | No |
| 165 | NKX2-8 | NK2 homeobox 8 [Source:HGNC Symbol;Acc:HGNC:16364] | 6826 | -0.065 | -0.2132 | No |
| 166 | ATF1 | activating transcription factor 1 [Source:HGNC Symbol;Acc:HGNC:783] | 6889 | -0.071 | -0.2175 | No |
| 167 | HOXB1 | homeobox B1 [Source:HGNC Symbol;Acc:HGNC:5111] | 6893 | -0.072 | -0.2171 | No |
| 168 | IKZF3 | IKAROS family zinc finger 3 [Source:HGNC Symbol;Acc:HGNC:13178] | 6905 | -0.073 | -0.2174 | No |
| 169 | BACH1 | BTB domain and CNC homolog 1 [Source:HGNC Symbol;Acc:HGNC:935] | 6909 | -0.073 | -0.2170 | No |
| 170 | PLSCR1 | phospholipid scramblase 1 [Source:HGNC Symbol;Acc:HGNC:9092] | 6917 | -0.073 | -0.2170 | No |
| 171 | BORCS8-MEF2B | BORCS8-MEF2B readthrough [Source:HGNC Symbol;Acc:HGNC:39979] | 7093 | -0.087 | -0.2302 | No |
| 172 | FEV | "FEV transcription factor, ETS family member [Source:HGNC Symbol;Acc:HGNC:18562]" | 7135 | -0.091 | -0.2327 | No |
| 173 | FOXN1 | forkhead box N1 [Source:HGNC Symbol;Acc:HGNC:12765] | 7140 | -0.092 | -0.2322 | No |
| 174 | ZNF574 | zinc finger protein 574 [Source:HGNC Symbol;Acc:HGNC:26166] | 7147 | -0.092 | -0.2319 | No |
| 175 | HNF4A | hepatocyte nuclear factor 4 alpha [Source:HGNC Symbol;Acc:HGNC:5024] | 7160 | -0.093 | -0.2321 | No |
| 176 | PITX3 | paired like homeodomain 3 [Source:HGNC Symbol;Acc:HGNC:9006] | 7197 | -0.096 | -0.2342 | No |
| 177 | TBX21 | T-box transcription factor 21 [Source:HGNC Symbol;Acc:HGNC:11599] | 7207 | -0.097 | -0.2341 | No |
| 178 | BCL11B | BAF chromatin remodeling complex subunit BCL11B [Source:HGNC Symbol;Acc:HGNC:13222] | 7260 | -0.100 | -0.2374 | No |
| 179 | POU3F2 | POU class 3 homeobox 2 [Source:HGNC Symbol;Acc:HGNC:9215] | 7302 | -0.102 | -0.2398 | No |
| 180 | CREB3L1 | cAMP responsive element binding protein 3 like 1 [Source:HGNC Symbol;Acc:HGNC:18856] | 7324 | -0.104 | -0.2406 | No |
| 181 | ETV2 | ETS variant transcription factor 2 [Source:HGNC Symbol;Acc:HGNC:3491] | 7365 | -0.108 | -0.2428 | No |
| 182 | TFCP2L1 | transcription factor CP2 like 1 [Source:HGNC Symbol;Acc:HGNC:17925] | 7398 | -0.111 | -0.2445 | No |
| 183 | SIX2 | SIX homeobox 2 [Source:HGNC Symbol;Acc:HGNC:10888] | 7417 | -0.112 | -0.2449 | No |
| 184 | LEF1 | lymphoid enhancer binding factor 1 [Source:HGNC Symbol;Acc:HGNC:6551] | 7467 | -0.116 | -0.2479 | No |
| 185 | CREB1 | cAMP responsive element binding protein 1 [Source:HGNC Symbol;Acc:HGNC:2345] | 7470 | -0.116 | -0.2471 | No |
| 186 | ZNF208 | zinc finger protein 208 [Source:HGNC Symbol;Acc:HGNC:12999] | 7487 | -0.117 | -0.2473 | No |
| 187 | ZNF473 | zinc finger protein 473 [Source:HGNC Symbol;Acc:HGNC:23239] | 7497 | -0.118 | -0.2471 | No |
| 188 | DMRT1 | doublesex and mab-3 related transcription factor 1 [Source:HGNC Symbol;Acc:HGNC:2934] | 7508 | -0.119 | -0.2468 | No |
| 189 | CDX4 | caudal type homeobox 4 [Source:HGNC Symbol;Acc:HGNC:1808] | 7519 | -0.120 | -0.2466 | No |
| 190 | TCF3 | transcription factor 3 [Source:HGNC Symbol;Acc:HGNC:11633] | 7521 | -0.120 | -0.2457 | No |
| 191 | CREB3L2 | cAMP responsive element binding protein 3 like 2 [Source:HGNC Symbol;Acc:HGNC:23720] | 7580 | -0.125 | -0.2493 | No |
| 192 | TFE3 | transcription factor binding to IGHM enhancer 3 [Source:HGNC Symbol;Acc:HGNC:11752] | 7624 | -0.129 | -0.2516 | No |
| 193 | NHLH2 | nescient helix-loop-helix 2 [Source:HGNC Symbol;Acc:HGNC:7818] | 7626 | -0.129 | -0.2506 | No |
| 194 | ZNF91 | zinc finger protein 91 [Source:HGNC Symbol;Acc:HGNC:13166] | 7629 | -0.129 | -0.2496 | No |
| 195 | NR2E1 | nuclear receptor subfamily 2 group E member 1 [Source:HGNC Symbol;Acc:HGNC:7973] | 7660 | -0.132 | -0.2509 | No |
| 196 | POU1F1 | POU class 1 homeobox 1 [Source:HGNC Symbol;Acc:HGNC:9210] | 7681 | -0.133 | -0.2514 | No |
| 197 | ALX1 | ALX homeobox 1 [Source:HGNC Symbol;Acc:HGNC:1494] | 7682 | -0.133 | -0.2503 | No |
| 198 | ELF1 | E74 like ETS transcription factor 1 [Source:HGNC Symbol;Acc:HGNC:3316] | 7715 | -0.135 | -0.2517 | No |
| 199 | NFIB | nuclear factor I B [Source:HGNC Symbol;Acc:HGNC:7785] | 7729 | -0.136 | -0.2516 | No |
| 200 | HOXC13 | homeobox C13 [Source:HGNC Symbol;Acc:HGNC:5125] | 7738 | -0.137 | -0.2510 | No |
| 201 | SOX10 | SRY-box transcription factor 10 [Source:HGNC Symbol;Acc:HGNC:11190] | 7788 | -0.141 | -0.2538 | No |
| 202 | MYOD1 | myogenic differentiation 1 [Source:HGNC Symbol;Acc:HGNC:7611] | 7839 | -0.143 | -0.2565 | No |
| 203 | FOXI1 | forkhead box I1 [Source:HGNC Symbol;Acc:HGNC:3815] | 7888 | -0.147 | -0.2591 | No |
| 204 | ELK3 | ETS transcription factor ELK3 [Source:HGNC Symbol;Acc:HGNC:3325] | 7921 | -0.149 | -0.2604 | No |
| 205 | NRL | neural retina leucine zipper [Source:HGNC Symbol;Acc:HGNC:8002] | 7936 | -0.150 | -0.2603 | No |
| 206 | ZNF267 | zinc finger protein 267 [Source:HGNC Symbol;Acc:HGNC:13060] | 7964 | -0.152 | -0.2611 | No |
| 207 | PLAGL1 | PLAG1 like zinc finger 1 [Source:HGNC Symbol;Acc:HGNC:9046] | 8025 | -0.157 | -0.2646 | No |
| 208 | E2F2 | E2F transcription factor 2 [Source:HGNC Symbol;Acc:HGNC:3114] | 8090 | -0.162 | -0.2683 | No |
| 209 | BARX2 | BARX homeobox 2 [Source:HGNC Symbol;Acc:HGNC:956] | 8122 | -0.164 | -0.2694 | No |
| 210 | STAT6 | signal transducer and activator of transcription 6 [Source:HGNC Symbol;Acc:HGNC:11368] | 8191 | -0.170 | -0.2734 | No |
| 211 | HAND2 | heart and neural crest derivatives expressed 2 [Source:HGNC Symbol;Acc:HGNC:4808] | 8211 | -0.172 | -0.2734 | No |
| 212 | AR | androgen receptor [Source:HGNC Symbol;Acc:HGNC:644] | 8217 | -0.173 | -0.2724 | No |
| 213 | TBX19 | T-box transcription factor 19 [Source:HGNC Symbol;Acc:HGNC:11596] | 8233 | -0.174 | -0.2721 | No |
| 214 | FOXD1 | forkhead box D1 [Source:HGNC Symbol;Acc:HGNC:3802] | 8236 | -0.174 | -0.2708 | No |
| 215 | HOXD10 | homeobox D10 [Source:HGNC Symbol;Acc:HGNC:5133] | 8257 | -0.176 | -0.2709 | No |
| 216 | ZNF268 | zinc finger protein 268 [Source:HGNC Symbol;Acc:HGNC:13061] | 8261 | -0.176 | -0.2696 | No |
| 217 | FOXJ3 | forkhead box J3 [Source:HGNC Symbol;Acc:HGNC:29178] | 8279 | -0.177 | -0.2695 | No |
| 218 | TEAD4 | TEA domain transcription factor 4 [Source:HGNC Symbol;Acc:HGNC:11717] | 8291 | -0.178 | -0.2689 | No |
| 219 | POU4F2 | POU class 4 homeobox 2 [Source:HGNC Symbol;Acc:HGNC:9219] | 8292 | -0.178 | -0.2674 | No |
| 220 | RAX | retina and anterior neural fold homeobox [Source:HGNC Symbol;Acc:HGNC:18662] | 8428 | -0.187 | -0.2766 | No |
| 221 | ZNF580 | zinc finger protein 580 [Source:HGNC Symbol;Acc:HGNC:29473] | 8433 | -0.188 | -0.2753 | No |
| 222 | E2F4 | E2F transcription factor 4 [Source:HGNC Symbol;Acc:HGNC:3118] | 8445 | -0.190 | -0.2746 | No |
| 223 | STAT3 | signal transducer and activator of transcription 3 [Source:HGNC Symbol;Acc:HGNC:11364] | 8453 | -0.190 | -0.2735 | No |
| 224 | NR1H4 | nuclear receptor subfamily 1 group H member 4 [Source:HGNC Symbol;Acc:HGNC:7967] | 8481 | -0.192 | -0.2740 | No |
| 225 | ATF2 | activating transcription factor 2 [Source:HGNC Symbol;Acc:HGNC:784] | 8592 | -0.201 | -0.2811 | No |
| 226 | VEZF1 | vascular endothelial zinc finger 1 [Source:HGNC Symbol;Acc:HGNC:12949] | 8621 | -0.202 | -0.2816 | No |
| 227 | MEIS3P1 | Meis homeobox 3 pseudogene 1 [Source:HGNC Symbol;Acc:HGNC:7002] | 8657 | -0.205 | -0.2827 | No |
| 228 | ATF6 | activating transcription factor 6 [Source:HGNC Symbol;Acc:HGNC:791] | 8680 | -0.208 | -0.2827 | No |
| 229 | ZBTB7B | zinc finger and BTB domain containing 7B [Source:HGNC Symbol;Acc:HGNC:18668] | 8702 | -0.209 | -0.2826 | No |
| 230 | NEUROD2 | neuronal differentiation 2 [Source:HGNC Symbol;Acc:HGNC:7763] | 8707 | -0.210 | -0.2811 | No |
| 231 | NR6A1 | nuclear receptor subfamily 6 group A member 1 [Source:HGNC Symbol;Acc:HGNC:7985] | 8725 | -0.211 | -0.2807 | No |
| 232 | RBPJ | recombination signal binding protein for immunoglobulin kappa J region [Source:HGNC Symbol;Acc:HGNC:5724] | 8800 | -0.217 | -0.2848 | No |
| 233 | TFAP2C | transcription factor AP-2 gamma [Source:HGNC Symbol;Acc:HGNC:11744] | 8824 | -0.219 | -0.2848 | No |
| 234 | HSF2 | heat shock transcription factor 2 [Source:HGNC Symbol;Acc:HGNC:5225] | 8845 | -0.221 | -0.2845 | No |
| 235 | TCF15 | transcription factor 15 [Source:HGNC Symbol;Acc:HGNC:11627] | 8925 | -0.226 | -0.2889 | No |
| 236 | NKX2-2 | NK2 homeobox 2 [Source:HGNC Symbol;Acc:HGNC:7835] | 8977 | -0.230 | -0.2910 | No |
| 237 | PRDM2 | PR/SET domain 2 [Source:HGNC Symbol;Acc:HGNC:9347] | 8992 | -0.231 | -0.2901 | No |
| 238 | SPI1 | Spi-1 proto-oncogene [Source:HGNC Symbol;Acc:HGNC:11241] | 9012 | -0.232 | -0.2897 | No |
| 239 | SMAD4 | SMAD family member 4 [Source:HGNC Symbol;Acc:HGNC:6770] | 9035 | -0.235 | -0.2895 | No |
| 240 | PGR | progesterone receptor [Source:HGNC Symbol;Acc:HGNC:8910] | 9048 | -0.235 | -0.2884 | No |
| 241 | RUNX1 | RUNX family transcription factor 1 [Source:HGNC Symbol;Acc:HGNC:10471] | 9063 | -0.237 | -0.2875 | No |
| 242 | CEBPE | CCAAT enhancer binding protein epsilon [Source:HGNC Symbol;Acc:HGNC:1836] | 9079 | -0.238 | -0.2867 | No |
| 243 | HAND1 | heart and neural crest derivatives expressed 1 [Source:HGNC Symbol;Acc:HGNC:4807] | 9082 | -0.238 | -0.2849 | No |
| 244 | DLX2 | distal-less homeobox 2 [Source:HGNC Symbol;Acc:HGNC:2915] | 9168 | -0.245 | -0.2896 | No |
| 245 | PHOX2B | paired like homeobox 2B [Source:HGNC Symbol;Acc:HGNC:9143] | 9179 | -0.246 | -0.2883 | No |
| 246 | POU2F3 | POU class 2 homeobox 3 [Source:HGNC Symbol;Acc:HGNC:19864] | 9181 | -0.246 | -0.2863 | No |
| 247 | TLX1 | T cell leukemia homeobox 1 [Source:HGNC Symbol;Acc:HGNC:5056] | 9200 | -0.248 | -0.2856 | No |
| 248 | ZNF24 | zinc finger protein 24 [Source:HGNC Symbol;Acc:HGNC:13032] | 9234 | -0.251 | -0.2861 | No |
| 249 | ZNF143 | zinc finger protein 143 [Source:HGNC Symbol;Acc:HGNC:12928] | 9246 | -0.252 | -0.2849 | No |
| 250 | SOX4 | SRY-box transcription factor 4 [Source:HGNC Symbol;Acc:HGNC:11200] | 9291 | -0.255 | -0.2862 | No |
| 251 | POU4F1 | POU class 4 homeobox 1 [Source:HGNC Symbol;Acc:HGNC:9218] | 9300 | -0.256 | -0.2847 | No |
| 252 | MAFB | MAF bZIP transcription factor B [Source:HGNC Symbol;Acc:HGNC:6408] | 9320 | -0.257 | -0.2840 | No |
| 253 | FOXJ1 | forkhead box J1 [Source:HGNC Symbol;Acc:HGNC:3816] | 9364 | -0.261 | -0.2852 | No |
| 254 | CRX | cone-rod homeobox [Source:HGNC Symbol;Acc:HGNC:2383] | 9397 | -0.263 | -0.2856 | No |
| 255 | ERG | ETS transcription factor ERG [Source:HGNC Symbol;Acc:HGNC:3446] | 9543 | -0.278 | -0.2948 | No |
| 256 | DDIT3 | DNA damage inducible transcript 3 [Source:HGNC Symbol;Acc:HGNC:2726] | 9551 | -0.278 | -0.2930 | No |
| 257 | MYF5 | myogenic factor 5 [Source:HGNC Symbol;Acc:HGNC:7565] | 9572 | -0.280 | -0.2922 | No |
| 258 | HNF1A | HNF1 homeobox A [Source:HGNC Symbol;Acc:HGNC:11621] | 9621 | -0.285 | -0.2936 | No |
| 259 | KLF15 | Kruppel like factor 15 [Source:HGNC Symbol;Acc:HGNC:14536] | 9635 | -0.286 | -0.2922 | No |
| 260 | IRF5 | interferon regulatory factor 5 [Source:HGNC Symbol;Acc:HGNC:6120] | 9688 | -0.290 | -0.2939 | No |
| 261 | PLAGL2 | PLAG1 like zinc finger 2 [Source:HGNC Symbol;Acc:HGNC:9047] | 9788 | -0.300 | -0.2993 | Yes |
| 262 | ETS1 | "ETS proto-oncogene 1, transcription factor [Source:HGNC Symbol;Acc:HGNC:3488]" | 9795 | -0.301 | -0.2972 | Yes |
| 263 | ZNF197 | zinc finger protein 197 [Source:HGNC Symbol;Acc:HGNC:12988] | 9800 | -0.301 | -0.2950 | Yes |
| 264 | PBX2 | PBX homeobox 2 [Source:HGNC Symbol;Acc:HGNC:8633] | 9804 | -0.302 | -0.2927 | Yes |
| 265 | NKX2-5 | NK2 homeobox 5 [Source:HGNC Symbol;Acc:HGNC:2488] | 9875 | -0.309 | -0.2956 | Yes |
| 266 | SATB2 | SATB homeobox 2 [Source:HGNC Symbol;Acc:HGNC:21637] | 9883 | -0.310 | -0.2936 | Yes |
| 267 | FOXH1 | forkhead box H1 [Source:HGNC Symbol;Acc:HGNC:3814] | 9884 | -0.310 | -0.2909 | Yes |
| 268 | SMAD1 | SMAD family member 1 [Source:HGNC Symbol;Acc:HGNC:6767] | 9905 | -0.312 | -0.2899 | Yes |
| 269 | HOXC11 | homeobox C11 [Source:HGNC Symbol;Acc:HGNC:5123] | 9907 | -0.312 | -0.2873 | Yes |
| 270 | TCF4 | transcription factor 4 [Source:HGNC Symbol;Acc:HGNC:11634] | 9918 | -0.313 | -0.2855 | Yes |
| 271 | FOXF1 | forkhead box F1 [Source:HGNC Symbol;Acc:HGNC:3809] | 9933 | -0.315 | -0.2839 | Yes |
| 272 | CSRNP3 | cysteine and serine rich nuclear protein 3 [Source:HGNC Symbol;Acc:HGNC:30729] | 9963 | -0.317 | -0.2836 | Yes |
| 273 | ELF4 | E74 like ETS transcription factor 4 [Source:HGNC Symbol;Acc:HGNC:3319] | 10071 | -0.328 | -0.2893 | Yes |
| 274 | MTF1 | metal regulatory transcription factor 1 [Source:HGNC Symbol;Acc:HGNC:7428] | 10125 | -0.334 | -0.2907 | Yes |
| 275 | CASZ1 | castor zinc finger 1 [Source:HGNC Symbol;Acc:HGNC:26002] | 10186 | -0.339 | -0.2926 | Yes |
| 276 | FOXD2 | forkhead box D2 [Source:HGNC Symbol;Acc:HGNC:3803] | 10209 | -0.341 | -0.2915 | Yes |
| 277 | PRDM16 | PR/SET domain 16 [Source:HGNC Symbol;Acc:HGNC:14000] | 10211 | -0.342 | -0.2887 | Yes |
| 278 | GCM1 | glial cells missing transcription factor 1 [Source:HGNC Symbol;Acc:HGNC:4197] | 10236 | -0.345 | -0.2877 | Yes |
| 279 | ZBTB16 | zinc finger and BTB domain containing 16 [Source:HGNC Symbol;Acc:HGNC:12930] | 10246 | -0.346 | -0.2855 | Yes |
| 280 | VENTX | VENT homeobox [Source:HGNC Symbol;Acc:HGNC:13639] | 10290 | -0.350 | -0.2859 | Yes |
| 281 | BATF | basic leucine zipper ATF-like transcription factor [Source:HGNC Symbol;Acc:HGNC:958] | 10312 | -0.352 | -0.2846 | Yes |
| 282 | GMEB1 | glucocorticoid modulatory element binding protein 1 [Source:HGNC Symbol;Acc:HGNC:4370] | 10328 | -0.354 | -0.2828 | Yes |
| 283 | MLXIP | MLX interacting protein [Source:HGNC Symbol;Acc:HGNC:17055] | 10357 | -0.357 | -0.2820 | Yes |
| 284 | ZNF224 | zinc finger protein 224 [Source:HGNC Symbol;Acc:HGNC:13017] | 10484 | -0.371 | -0.2889 | Yes |
| 285 | KLF10 | Kruppel like factor 10 [Source:HGNC Symbol;Acc:HGNC:11810] | 10631 | -0.386 | -0.2973 | Yes |
| 286 | FOS | "Fos proto-oncogene, AP-1 transcription factor subunit [Source:HGNC Symbol;Acc:HGNC:3796]" | 10640 | -0.387 | -0.2947 | Yes |
| 287 | TFAP2B | transcription factor AP-2 beta [Source:HGNC Symbol;Acc:HGNC:11743] | 10643 | -0.387 | -0.2916 | Yes |
| 288 | LITAF | lipopolysaccharide induced TNF factor [Source:HGNC Symbol;Acc:HGNC:16841] | 10649 | -0.388 | -0.2887 | Yes |
| 289 | RBPJL | recombination signal binding protein for immunoglobulin kappa J region like [Source:HGNC Symbol;Acc:HGNC:13761] | 10650 | -0.388 | -0.2854 | Yes |
| 290 | CSRNP2 | cysteine and serine rich nuclear protein 2 [Source:HGNC Symbol;Acc:HGNC:16006] | 10669 | -0.390 | -0.2835 | Yes |
| 291 | IRF1 | interferon regulatory factor 1 [Source:HGNC Symbol;Acc:HGNC:6116] | 10700 | -0.393 | -0.2826 | Yes |
| 292 | SOX17 | SRY-box transcription factor 17 [Source:HGNC Symbol;Acc:HGNC:18122] | 10843 | -0.410 | -0.2905 | Yes |
| 293 | ELK4 | ETS transcription factor ELK4 [Source:HGNC Symbol;Acc:HGNC:3326] | 10862 | -0.411 | -0.2884 | Yes |
| 294 | IRF3 | interferon regulatory factor 3 [Source:HGNC Symbol;Acc:HGNC:6118] | 10879 | -0.414 | -0.2862 | Yes |
| 295 | GATA1 | GATA binding protein 1 [Source:HGNC Symbol;Acc:HGNC:4170] | 10958 | -0.425 | -0.2888 | Yes |
| 296 | FOXF2 | forkhead box F2 [Source:HGNC Symbol;Acc:HGNC:3810] | 10975 | -0.427 | -0.2865 | Yes |
| 297 | ZBTB17 | zinc finger and BTB domain containing 17 [Source:HGNC Symbol;Acc:HGNC:12936] | 10996 | -0.429 | -0.2844 | Yes |
| 298 | RFX2 | regulatory factor X2 [Source:HGNC Symbol;Acc:HGNC:9983] | 11051 | -0.438 | -0.2850 | Yes |
| 299 | GATA2 | GATA binding protein 2 [Source:HGNC Symbol;Acc:HGNC:4171] | 11058 | -0.439 | -0.2818 | Yes |
| 300 | PRDM4 | PR/SET domain 4 [Source:HGNC Symbol;Acc:HGNC:9348] | 11098 | -0.443 | -0.2811 | Yes |
| 301 | PPARA | peroxisome proliferator activated receptor alpha [Source:HGNC Symbol;Acc:HGNC:9232] | 11110 | -0.445 | -0.2783 | Yes |
| 302 | NRF1 | nuclear respiratory factor 1 [Source:HGNC Symbol;Acc:HGNC:7996] | 11141 | -0.449 | -0.2768 | Yes |
| 303 | MEF2D | myocyte enhancer factor 2D [Source:HGNC Symbol;Acc:HGNC:6997] | 11149 | -0.451 | -0.2736 | Yes |
| 304 | EGR3 | early growth response 3 [Source:HGNC Symbol;Acc:HGNC:3240] | 11207 | -0.458 | -0.2743 | Yes |
| 305 | FOSL1 | "FOS like 1, AP-1 transcription factor subunit [Source:HGNC Symbol;Acc:HGNC:13718]" | 11222 | -0.460 | -0.2715 | Yes |
| 306 | NR2C2 | nuclear receptor subfamily 2 group C member 2 [Source:HGNC Symbol;Acc:HGNC:7972] | 11257 | -0.465 | -0.2702 | Yes |
| 307 | NEUROD6 | neuronal differentiation 6 [Source:HGNC Symbol;Acc:HGNC:13804] | 11258 | -0.465 | -0.2663 | Yes |
| 308 | FOXO3 | forkhead box O3 [Source:HGNC Symbol;Acc:HGNC:3821] | 11305 | -0.472 | -0.2660 | Yes |
| 309 | NR5A2 | nuclear receptor subfamily 5 group A member 2 [Source:HGNC Symbol;Acc:HGNC:7984] | 11362 | -0.481 | -0.2664 | Yes |
| 310 | ZFX | zinc finger protein X-linked [Source:HGNC Symbol;Acc:HGNC:12869] | 11372 | -0.482 | -0.2630 | Yes |
| 311 | PROP1 | PROP paired-like homeobox 1 [Source:HGNC Symbol;Acc:HGNC:9455] | 11373 | -0.482 | -0.2589 | Yes |
| 312 | ZNF384 | zinc finger protein 384 [Source:HGNC Symbol;Acc:HGNC:11955] | 11385 | -0.483 | -0.2557 | Yes |
| 313 | KLF13 | Kruppel like factor 13 [Source:HGNC Symbol;Acc:HGNC:13672] | 11594 | -0.515 | -0.2680 | Yes |
| 314 | FOXK2 | forkhead box K2 [Source:HGNC Symbol;Acc:HGNC:6036] | 11609 | -0.517 | -0.2647 | Yes |
| 315 | ZNF76 | zinc finger protein 76 [Source:HGNC Symbol;Acc:HGNC:13149] | 11651 | -0.525 | -0.2635 | Yes |
| 316 | NR4A1 | nuclear receptor subfamily 4 group A member 1 [Source:HGNC Symbol;Acc:HGNC:7980] | 11709 | -0.535 | -0.2635 | Yes |
| 317 | EPAS1 | endothelial PAS domain protein 1 [Source:HGNC Symbol;Acc:HGNC:3374] | 11758 | -0.542 | -0.2628 | Yes |
| 318 | UBP1 | upstream binding protein 1 [Source:HGNC Symbol;Acc:HGNC:12507] | 11773 | -0.545 | -0.2593 | Yes |
| 319 | HIF1A | hypoxia inducible factor 1 subunit alpha [Source:HGNC Symbol;Acc:HGNC:4910] | 11833 | -0.556 | -0.2593 | Yes |
| 320 | RXRB | retinoid X receptor beta [Source:HGNC Symbol;Acc:HGNC:10478] | 11871 | -0.565 | -0.2574 | Yes |
| 321 | REL | "REL proto-oncogene, NF-kB subunit [Source:HGNC Symbol;Acc:HGNC:9954]" | 11915 | -0.575 | -0.2560 | Yes |
| 322 | ATF4 | activating transcription factor 4 [Source:HGNC Symbol;Acc:HGNC:786] | 11999 | -0.593 | -0.2576 | Yes |
| 323 | PAX9 | paired box 9 [Source:HGNC Symbol;Acc:HGNC:8623] | 12028 | -0.600 | -0.2548 | Yes |
| 324 | EGR1 | early growth response 1 [Source:HGNC Symbol;Acc:HGNC:3238] | 12052 | -0.607 | -0.2515 | Yes |
| 325 | MSX1 | msh homeobox 1 [Source:HGNC Symbol;Acc:HGNC:7391] | 12054 | -0.609 | -0.2464 | Yes |
| 326 | GABPA | GA binding protein transcription factor subunit alpha [Source:HGNC Symbol;Acc:HGNC:4071] | 12117 | -0.624 | -0.2460 | Yes |
| 327 | GMEB2 | glucocorticoid modulatory element binding protein 2 [Source:HGNC Symbol;Acc:HGNC:4371] | 12135 | -0.628 | -0.2421 | Yes |
| 328 | ATF6B | activating transcription factor 6 beta [Source:HGNC Symbol;Acc:HGNC:2349] | 12171 | -0.636 | -0.2395 | Yes |
| 329 | CEBPD | CCAAT enhancer binding protein delta [Source:HGNC Symbol;Acc:HGNC:1835] | 12216 | -0.650 | -0.2375 | Yes |
| 330 | FOXC2 | forkhead box C2 [Source:HGNC Symbol;Acc:HGNC:3801] | 12218 | -0.650 | -0.2321 | Yes |
| 331 | TEF | "TEF transcription factor, PAR bZIP family member [Source:HGNC Symbol;Acc:HGNC:11722]" | 12228 | -0.653 | -0.2273 | Yes |
| 332 | YY1 | YY1 transcription factor [Source:HGNC Symbol;Acc:HGNC:12856] | 12229 | -0.653 | -0.2217 | Yes |
| 333 | ETV6 | ETS variant transcription factor 6 [Source:HGNC Symbol;Acc:HGNC:3495] | 12231 | -0.654 | -0.2163 | Yes |
| 334 | FOXO4 | forkhead box O4 [Source:HGNC Symbol;Acc:HGNC:7139] | 12240 | -0.656 | -0.2113 | Yes |
| 335 | KLF7 | Kruppel like factor 7 [Source:HGNC Symbol;Acc:HGNC:6350] | 12271 | -0.669 | -0.2081 | Yes |
| 336 | ALX4 | ALX homeobox 4 [Source:HGNC Symbol;Acc:HGNC:450] | 12273 | -0.670 | -0.2025 | Yes |
| 337 | GATA3 | GATA binding protein 3 [Source:HGNC Symbol;Acc:HGNC:4172] | 12276 | -0.670 | -0.1970 | Yes |
| 338 | NFIA | nuclear factor I A [Source:HGNC Symbol;Acc:HGNC:7784] | 12294 | -0.675 | -0.1926 | Yes |
| 339 | ATF5 | activating transcription factor 5 [Source:HGNC Symbol;Acc:HGNC:790] | 12310 | -0.680 | -0.1880 | Yes |
| 340 | ETV4 | ETS variant transcription factor 4 [Source:HGNC Symbol;Acc:HGNC:3493] | 12311 | -0.680 | -0.1823 | Yes |
| 341 | MYOG | myogenin [Source:HGNC Symbol;Acc:HGNC:7612] | 12313 | -0.681 | -0.1766 | Yes |
| 342 | GLI1 | GLI family zinc finger 1 [Source:HGNC Symbol;Acc:HGNC:4317] | 12333 | -0.687 | -0.1723 | Yes |
| 343 | NR4A2 | nuclear receptor subfamily 4 group A member 2 [Source:HGNC Symbol;Acc:HGNC:7981] | 12368 | -0.698 | -0.1691 | Yes |
| 344 | ZNF292 | zinc finger protein 292 [Source:HGNC Symbol;Acc:HGNC:18410] | 12377 | -0.702 | -0.1638 | Yes |
| 345 | JUNB | "JunB proto-oncogene, AP-1 transcription factor subunit [Source:HGNC Symbol;Acc:HGNC:6205]" | 12468 | -0.734 | -0.1648 | Yes |
| 346 | SOX2 | SRY-box transcription factor 2 [Source:HGNC Symbol;Acc:HGNC:11195] | 12485 | -0.742 | -0.1597 | Yes |
| 347 | FOSL2 | "FOS like 2, AP-1 transcription factor subunit [Source:HGNC Symbol;Acc:HGNC:3798]" | 12510 | -0.751 | -0.1553 | Yes |
| 348 | MZF1 | myeloid zinc finger 1 [Source:HGNC Symbol;Acc:HGNC:13108] | 12528 | -0.762 | -0.1502 | Yes |
| 349 | ZNF814 | zinc finger protein 814 [Source:HGNC Symbol;Acc:HGNC:33258] | 12537 | -0.765 | -0.1444 | Yes |
| 350 | NR4A3 | nuclear receptor subfamily 4 group A member 3 [Source:HGNC Symbol;Acc:HGNC:7982] | 12543 | -0.769 | -0.1382 | Yes |
| 351 | SRF | serum response factor [Source:HGNC Symbol;Acc:HGNC:11291] | 12549 | -0.773 | -0.1321 | Yes |
| 352 | SP1 | Sp1 transcription factor [Source:HGNC Symbol;Acc:HGNC:11205] | 12557 | -0.777 | -0.1261 | Yes |
| 353 | FOSB | "FosB proto-oncogene, AP-1 transcription factor subunit [Source:HGNC Symbol;Acc:HGNC:3797]" | 12571 | -0.782 | -0.1205 | Yes |
| 354 | RREB1 | ras responsive element binding protein 1 [Source:HGNC Symbol;Acc:HGNC:10449] | 12576 | -0.784 | -0.1142 | Yes |
| 355 | MAFG | MAF bZIP transcription factor G [Source:HGNC Symbol;Acc:HGNC:6781] | 12584 | -0.785 | -0.1081 | Yes |
| 356 | ATF3 | activating transcription factor 3 [Source:HGNC Symbol;Acc:HGNC:785] | 12659 | -0.833 | -0.1069 | Yes |
| 357 | KLF5 | Kruppel like factor 5 [Source:HGNC Symbol;Acc:HGNC:6349] | 12672 | -0.845 | -0.1007 | Yes |
| 358 | NFIC | nuclear factor I C [Source:HGNC Symbol;Acc:HGNC:7786] | 12697 | -0.862 | -0.0953 | Yes |
| 359 | NFAT5 | nuclear factor of activated T cells 5 [Source:HGNC Symbol;Acc:HGNC:7774] | 12741 | -0.903 | -0.0911 | Yes |
| 360 | NFKB2 | nuclear factor kappa B subunit 2 [Source:HGNC Symbol;Acc:HGNC:7795] | 12750 | -0.912 | -0.0840 | Yes |
| 361 | KLF6 | Kruppel like factor 6 [Source:HGNC Symbol;Acc:HGNC:2235] | 12752 | -0.915 | -0.0764 | Yes |
| 362 | HOXC4 | homeobox C4 [Source:HGNC Symbol;Acc:HGNC:5126] | 12764 | -0.924 | -0.0694 | Yes |
| 363 | MAFF | MAF bZIP transcription factor F [Source:HGNC Symbol;Acc:HGNC:6780] | 12766 | -0.925 | -0.0616 | Yes |
| 364 | MYC | "MYC proto-oncogene, bHLH transcription factor [Source:HGNC Symbol;Acc:HGNC:7553]" | 12801 | -0.970 | -0.0561 | Yes |
| 365 | CEBPB | CCAAT enhancer binding protein beta [Source:HGNC Symbol;Acc:HGNC:1834] | 12804 | -0.973 | -0.0481 | Yes |
| 366 | JUN | "Jun proto-oncogene, AP-1 transcription factor subunit [Source:HGNC Symbol;Acc:HGNC:6204]" | 12813 | -0.994 | -0.0403 | Yes |
| 367 | RLF | RLF zinc finger [Source:HGNC Symbol;Acc:HGNC:10025] | 12836 | -1.040 | -0.0332 | Yes |
| 368 | KLF4 | Kruppel like factor 4 [Source:HGNC Symbol;Acc:HGNC:6348] | 12848 | -1.058 | -0.0251 | Yes |
| 369 | EBF2 | EBF transcription factor 2 [Source:HGNC Symbol;Acc:HGNC:19090] | 12859 | -1.088 | -0.0167 | Yes |
| 370 | JUND | "JunD proto-oncogene, AP-1 transcription factor subunit [Source:HGNC Symbol;Acc:HGNC:6206]" | 12865 | -1.116 | -0.0077 | Yes |
| 371 | E2F3 | E2F transcription factor 3 [Source:HGNC Symbol;Acc:HGNC:3115] | 12886 | -1.224 | 0.0011 | Yes |
Table: GSEA details [plain text format]

  

Fig 2: GOMF\_DNA\_BINDING\_TRANSCRIPTION\_ACTIVATOR\_ACTIVITY      
 Blue-Pink O' Gram in the Space of the Analyzed GeneSet

  

Fig 3: GOMF\_DNA\_BINDING\_TRANSCRIPTION\_ACTIVATOR\_ACTIVITY: Random ES distribution      
 Gene set null distribution of ES for **GOMF\_DNA\_BINDING\_TRANSCRIPTION\_ACTIVATOR\_ACTIVITY**
